# Collaboration, cooperation, communication, contact and competencies 

**DOI:** 10.3205/zma001036

**Published:** 2016-04-29

**Authors:** Jill E. Thistlethwaite

**Affiliations:** 1 University of Technology Sydney, Health professions education consultant, Adjunct professor, Sydney Australia

## Introduction

The global interprofessional education (IPE) community is expanding. However too frequently native English speakers like myself confine ourselves to literature in English and have little awareness of what has been going on for some time in countries under-represented in the English language journals. This deficiency has been highlighted for me not only by the richness of the papers in this special edition of the GMS Journal for Medical Education but also by my recent experiences co-editing a series of books on leadership for IPE and my travels outside Australia and the UK. The books include chapters from Indonesia, Japan, Malaysia, India, the Philippines, Kenya and South Africa as well as the more frequently published countries such as the US, UK, Australia, New Zealand and Canada [1], [2], [3]. 

## Competencies

I was honoured to be asked to write the editorial for this special edition and will share some thoughts on the current state of IPE from my perspective on evaluation and what we need to do to ensure sustainability of initiatives in the future. Perhaps not surprisingly, I was struck by the similarities in focus of the German projects compared to other locations. Five c-words stood out (in their English translation): collaboration; cooperation; communication; contact; and competencies. The first four of these can be thought of as examples of the intended outcomes of IPE and interprofessional practice (IPP) and appear frequently in the papers in this collection. The last word reflects the competency-based movement prevalent within medical and other health professions education. A number of bodies have defined the competencies they feel are required for IPP that may be developed through IPE, for example the Canadian Interprofessional Health Collaborative (CIHC) [http:// www.cihc.ca/files/CIHC_IPCompetencies_Feb1210.pdf], and the Interprofessional Education Collaborative (IPEC in the US) [http:// www.aacn.nche.edu/education-resources/ipecreport.pdf]. However, there has also been criticism that competencies reduce complex activities and tasks to simple checklists that over simplify the concepts involved [4]. The complexity of IPP and collaborative practice is reflected in the number of competencies listed in frequently referenced frameworks [5]. This is similar to ‘communication’ as Bachmann et al. discuss in their paper, which focuses on the translation of the Health Professions Core Communication Curriculum (HPCCC) from German into English – a curriculum that has 61 learning objectives [6]. Higher education institutions (HEIs) that have health and social care professionals programmes and wish to introduce or expand interprofessional learning opportunities need to decide on the appropriate learning outcomes or competencies for such programmes. Educators should take into account the standards of the national accreditation bodies for each health profession at pre-certification (pre-licensure) level and use these to help define their outcomes. German HEIs may look to other countries for additional suitable examples but should base their work on the context of their own health services, health and social care professional mix and cultural imperatives. An example of the importance of context is shown in the paper by Eich-Krohm et al. In Germany nursing is not a university level programme, unlike some countries, and therefore nursing and medical students are less likely to interact during training unless specific opportunities are created for them to do so [7]. Bohrer and colleagues describe the Berlin project and the need to take into account differing university regulations in relation to education and assessment [8]. To be successful IPE needs to be integrated into curricula and the organization needs to be committed enough to provide resources such as space [9].

## Assessment

Once we can agree on competencies as an outcome of our curricula then we have to consider how these may be assessed, given that the majority of students only consider an activity is important if it is examined in some way: assessment “powerfully frames how students learn and what students achieve” [http:// www.assessmentfutures.com]. I certainly agree that learners need to know what they are expected to learn. While I am concerned about “competence” as the aim of education, it is also worrying that some interprofessional learning interventions do not have defined outcomes so learners are unsure what they are expected to learn [10]]. Within IPE this learner confusion may extend to why they have been placed with other health professionals when they want to focus on gaining their own profession’s knowledge. 

Sometimes IPE seems so logistically difficult and interprofessional educators so unsupported that we forget basic educational principles such as curriculum alignment [11]. Certainly in Australia our recent survey showed that most IPE was not assessed other than by attendance [http://www.hwa.gov.au/sites/uploads/IPE%20Audit%20report%20Jan%202013.pdf]. More in-depth assessment should be accompanied by observation and feedback so that assessment is itself a learning activity: assessment for learning rather than solely assessment of learning. Lack of assessment, once we have agreed what to assess, hampers our ability to answer that frequently asked question: “what is the evidence for IPE?” This question is about effectiveness and outcomes, and may have the sub-text of “why should we change what we do?” If we want to know whether IPE works, then we have to define what we mean by “works”. Proponents of the widely referenced modified Kirkpatrick model of educational outcomes [12] suggest that we ultimately we need to show that IPE leads to improved organizational (4a) or patient outcomes (4b). This is somewhat unfair given that rarely, if at all, do we expect other pre-qualification education in the early years of training, to demonstrate a difference in how organisations function, whether patients’ conditions improve or patient safety is enhanced. The Institute of Medicine in the US has suggested that what education can achieve, and what can be measured, is that learners meet learning outcomes that have been developed as applicable to optimal health care delivery that improves outcomes [13]. Trying to show a direct causal link between one aspect of a curriculum, such as IPE, and longer term effects is, in my opinion, impossible. What we should be able to do is evaluate whether learners have achieved those learning outcomes that we consider to be important for collaborative practice and are ready to practice in this way when they graduate. This assumes we have a clear idea, through research, of what those learning outcomes actually should be. So learning affects practice and practice affects outcomes. As with all pre-certification education we have to remember that learning does not stop on graduation and skills are developed throughout one’s practice through experience, feedback and reflection.

## Evaluation

Even if the Kirkpatrick model is adopted to plan evaluation, most published evaluations of IPE are still confined to learner reaction, attitudinal change and knowledge acquisition rather than looking at performance in the learning and working spaces [14]. Again this is not surprising given the difficulties of work-based observation and assessment. Learner satisfaction is important and necessary but not sufficient as evidence of utility. Overwhelmingly students appear to enjoy IPE and rate interprofessional experiences highly though there is great variation in the format, involvement, location and timing of IPE across programs, institutions, regions and countries. Berger et al. from Heidelberg have evaluated a newly introduced interprofessional seminar and shown that students who learn and work interprofessionally rate the experience more positively than students who are in uniprofessional groups [15]. Flentje et al. evaluated an interprofessional simulation exercise; participants self-reported that they had been able to improve their teamwork competencies particularly in relation to communication [16]. The University of Greifswald Medical School has also introduced a simulation exercise focused on a clinical emergency for medical and nursing students, and other relevant professions, which participants rated highly [17]. Meanwhile in Mannheim, medical and physiotherapy students have been learning in teams, with some difference of opinion between the two groups as to the outcomes [18]. As medical students and physiotherapy students have common competencies in rheumatic and musculoskeletal diseases these have formed the basis for successful implementation of interprofessional learning for these two groups [19].

With regards to attitudes, the commonly used tools, such as *RIPLS* (readiness for interprofessional learning scale) [20] and IEPS (interdisciplinary education perception scale) [21] have rarely shown change in more recent publications when applied before and after an intervention. Students start with positive attitudes, either because they have volunteered for elective interprofessional experiences or because IPE is now a more acceptable curricular inclusion. 

We may and should be critical of an approach that focuses solely on outcomes. Calls to evaluate and research IPE and IPP within theoretical frameworks [22], [23] to enhance scholarship and provide context highlight the need to explore the nature of IPE and IPP and the interactions between them. So far only a minority of evaluation studies have made specific reference to theories informing their approach, although adult learning theory continues to be frequently implicit (as noted ten years ago by Barr et al., 2005 [24]). When theories are invoked they are drawn from a diversity of disciplines including education, psychology and, particularly, sociology [25]. For example Hean et al. have foregrounded socio-cultural theory with its focus on the social aspect of learning (‘with, from and about) in their recommendations of theories relevant for IPE [26]. In this journal, with another focus on communication, Posenau and Peters draw on linguistics to analyse professional markers through a qualitative approach to the conversations that take place during interprofessional simulation activities [27].

## Realist evaluation

Evaluation of outcomes presupposes that there is a linear causality between input and output. However the space between input and output has been referred to as the “black box” [28] and it is seldom apparent from quantitative approaches what is going on in that box. Why do some students develop skills in teamwork and others not when the intervention has been the same? How do educators ensure that students achieve the same defined learning outcomes from clinical programmes, which offer different experiences depending on location, preceptor, length, timing and access to patients? Moreover the complexity of health professional education initiatives is such that they are rarely binary: neither “effective” or “ineffective”, and students are rarely “competent” or “not competent” to perform complex tasks, even if we can describe what competence looks like. 

A common type of paper in the interprofessional literature describes a learning activity that involves students from three to four professions interviewing a patient about the patient’s experience of living with a long term condition. Each student will have learnt a profession specific approach to “taking a history”. By observing each other’s language and questions, and then discussing the similarities and differences in approach, the objective is that students gain an understanding of each other’s roles in relation to a particular health care problem. Suppose that prior to the activity students have a quiz on professional roles, which is repeated two weeks after the exercise. Students are also asked to rate, before and after, their confidence in working with other professionals. Student marks indicate that 85% have increased their knowledge but the other 15% have not; 70% rate their confidence as improved, 20% as the same and 10% as worsened. These figures would suggest that overall the activity has been effective as the majority has changed in a positive way. However, we cannot know from this outcome why certain students learnt and others did not, why some feel more confident and others less confident. We may hypothesise a number of reasons for the discrepancy: differences in student motivation or engagement; lack of preparation; variations in the patient experiences; the professional mix of the groups; facilitator variability; the method of assessment; etc. We also know that student self-assessment of confidence is a poor measure of change and some students are better at this than others.

To explore the reasons for these outcomes we need to carry out some form of process evaluation in order to explore possible factors affecting effectiveness. Realist evaluation is a form of process evaluation which aims to answer the question: what works, for whom, in what circumstances, in what respects, to what extent and why [29]? Such an approach is time-consuming and therefore rarely done unless funded adequately – which programme evaluation rarely is. Realist methods involve in-depth case studies and reflexive questioning about why on this occasion a particular input produced a particular outcome but on a different occasion it produced the opposite outcome [30]. This type of evaluation was originally developed to explore complex social interventions such as health promotions campaigns for safe sex practices that depend on how different people respond to the same input to generate the anticipated outcomes [31]. 

Realist evaluation has been advocated as a useful method in medical education because of the complexity of the interventions involved [32]. What appears effective as an IPE programme in one institution may be disastrous in another because of contextual differences that are not always apparent or considered. For example the German context is different from some other European countries in that nursing is not a university-based program; how may this difference affect student learning? 

Realism is a philosophy of science situated between positivism and relativism/constructivism [33]. Realists disagree with the positivist stance on the nature of causality with its conflation of simple observations and descriptions of what happens with explanations [34]. The realist evaluator aims to elucidate underlying causal mechanisms and how they are affected by context in bringing about outcomes: context (C) + mechanism (M) = outcome (O) [35]. Here a mechanism is defined as ‘an underlying entity, process or structure which operates in particular contexts to generate outcomes of interest’ [28]. 

## Conclusion

If we cannot provide some explanation of what is happening in the “black box” of IPE and lack evidence of effectiveness in relation to the achievement of IPL outcomes, including collaboration, cooperation, and communication, we are going to have problems making a good case for sustaining IPE within institutions. The curricula of all health professions programmes are bulging as medical science knowledge expands, and calls are made for including new courses on, for example, professionalism, resilience and leadership. Funding for higher education is shrinking in many countries or student numbers are increased without additional resources being provided. As educators, clinical academics and interprofessional champions we need to build high quality evaluation and research into our courses. We need to be able to provide some answers to the questions about evidence – even if the questions are not framed in quite the way we would like them to be. The papers in this collection add to the literature that is required and should be required reading for all health professional educators in Germany. 

## Competing interests

The author declares that she has no competing interests.
